# Author's reply

**Published:** 2010

**Authors:** Siraj Wali

**Affiliations:** *College of Medicine, King Abdulaziz University Jeddah, Saudi Arabia E-mail: bintashfeen@yahoo.com*

Dear Editor;

First, I would like to thank the author for his comment regarding the case report of *Mycobacterium chelonae* empiema with bronchopleural fistula in an immunocompetent patient. I totally agree with his comment. Our patient may well have had MSMD, however, I wish there is a screening test for Interferon gamma receptor deficiencies. Diagnosis MSMD is established by flow cytometric evaluation of cell surface receptor expression on monocytes and lymphocytes as well as examining signaling either by intracellular staining (lack of STAT1 phosphorylation in response to IFN-gamma) or western blot. Detection of the mutation at the molecular level confirms the diagnosis. In my view, tell a screening test is available such cases will continue to be missed since not all institutions are capable of running the diagnostic test.

